# Functional role of DNA mismatch repair gene PMS2 in prostate cancer cells

**DOI:** 10.18632/oncotarget.3854

**Published:** 2015-05-06

**Authors:** Shinichiro Fukuhara, Inik Chang, Yozo Mitsui, Takeshi Chiyomaru, Soichiro Yamamura, Shahana Majid, Sharanjot Saini, Guoren Deng, Ankurpreet Gill, Darryn K. Wong, Hiroaki Shiina, Norio Nonomura, Yun-Fai C. Lau, Rajvir Dahiya, Yuichiro Tanaka

**Affiliations:** ^1^ Department of Surgery/Urology, Veterans Affairs Medical Center, San Francisco, California 94121, United States of America; ^2^ Department of Urology, University of California, San Francisco, California 94121, United States of America; ^3^ Department of Urology, Osaka University Graduate School of Medicine, Suita 565-0871, Japan; ^4^ Department of Oral Biology, Yonsei University College of Dentistry, Seoul 120-752, South Korea; ^5^ Department of Urology, Shimane University Faculty of Medicine, Izumo 693-8501, Japan; ^6^ Department of Urology, Kagoshima University Graduate School of Medical and Dental Sciences, Kagoshima 890-8544, Japan; ^7^ Department of Medicine, Veterans Affairs Medical Center, San Francisco, California 94121, United States of America; ^8^ Institute for Human Genetics, University of California, San Francisco, California 94121, United States of America

**Keywords:** PMS2, apoptosis, prostate cancer

## Abstract

DNA mismatch repair (MMR) enzymes act as proofreading complexes that maintains genomic integrity and MMR-deficient cells show an increased mutation rate. MMR has also been shown to influence cell signaling and the regulation of tumor development. MMR consists of various genes and includes post-meiotic segregation (PMS) 2 which is a vital component of mutL-alpha. In prostate, the functional role of this gene has never been reported and in this study, our aim was to investigate the effect of PMS2 on growth properties of prostate cancer (PCa) cells. Previous studies have shown PMS2 to be deficient in DU145 cells and this lack of expression was confirmed by Western blotting whereas normal prostatic PWR-1E and RWPE-1 cells expressed this gene. PMS2 effects on various growth properties of DU145 were then determined by creating stable gene transfectants. Interestingly, PMS2 caused decreased cell proliferation, migration, invasion, and *in vivo* growth; and increased apoptosis as compared to vector control. We further analyzed genes affected by PMS2 expression and observe the apoptosis-related TMS1 gene to be significantly upregulated whereas anti-apoptotic BCL2A1 was downregulated. These results demonstrate a functional role for PMS2 to protect against PCa progression by enhancing apoptosis of PCa cells.

## INTRODUCTION

Prostate cancer (PCa) ranked as the most frequently diagnosed malignancy among men in the United States of America with an estimated 233, 000 new incidences in the Year 2014 [1]. With an annual mortality rate of 29, 480, this malignancy ranked second in cancer deaths [1]. As an aging disease, invasive PCa is expected to occur in 1 in 43 of those aged 50 to 59, 1 in 16 aged 60 to 69, and 1 in 9 aged 70 years and older [1]. PCa is generally treatable with androgen-deprivation therapy however there is a high recurrence to an androgen-independent and metastatic state of this cancer that leads to death within several years. There are no effective therapies currently to cure androgen-independent PCa and thus, new prognostic markers and effective treatment strategies are desired.

A factor that can lead to PCa is DNA mismatch mutations and to overcome their harmful effects, the cell has the DNA mismatch repair (MMR) system that is able to correct these lesions. Deficiencies in the MMR pathway can result in higher rates of mutation or genetic instability that can cause defects in genes that regulate cell proliferation and death [2], thereby increasing susceptibility to cancers. Alternatively, MMR genes are capable of sensitizing cells toward DNA lesions and trigger apoptosis, cell cycle arrest, and cell death [3, 4]. Thus, MMR is vital for proper cellular function and health of the individual.

Among diseases known for mutations in MMR is hereditary non-polyposis colorectal cancer (HNPCC) and interestingly, clinical reports have shown PCa to arise from men afflicted with HNPCC [5, 6]. In fact, the lifetime risk of PCa was calculated to be double among individuals with HNPCC and defective MMR as compared to the general population [6]. In a study conducted in Norway, men described as carriers or obligate carriers of MMR mutation developed PCa at a significantly higher rate than that expected to occur by chance in the population [7]. These men were significantly younger at the time of diagnosis and with higher Gleason scores of 8 to 10 compared to men diagnosed prior to 70 years of age in the population. Further analysis calculated the cumulative risk of PCa by age 70 years to be 30% in MMR gene mutation carriers compared to 8% in the population. In another study evaluating a colon cancer family registry of 764 carriers of MMR gene mutations, a higher risk for PCa was observed compared to the general population with a standardized incidence ratio of 2.05 and 95% confidence interval of 1.23 to 3.01 [8]. Thus deficiencies of the MMR process can lead to PCa.

The MMR system consists of various proteins and among the MutL homologues is the post-meiotic segregation (PMS2) gene [9, 10]. PMS2 is located in chromosome 7p22 region, consists of 15 exons and is roughly 16 kilobases in length [11]. PMS2 is a nuclear protein that is composed of 862 amino acids with a size of 95.8 kDa. PMS2 heterodimerizes with MLH1 to form MutL alpha and together with MutS heterodimers (alpha or beta) and other co-factors, repairs DNA mismatches and insertion-deletion mispairs formed during DNA replication or recombination [10, 12]. PMS2 appears to have endonucleolytic activity during the repair process as Kadyrov et al. [13] identified a motif responsible for this role.

Hundreds of mutations and polymorphisms have been identified among the various MMR genes and includes the PMS2 gene [14]. Interestingly though not significant, PMS2 mutation carriers were observed to have more extra-colonic cancers [15]. In prostate, defects of PMS2 in cancer cell lines have been documented in prior studies. Among the first reports was the PCa line DU145 where Boyer et al. [16] identified a missense mutation in the PMS2 gene after demonstrating reduced MMR activity and microsatellite instability, an indicator of MMR deficiency. Chen et al. [17] confirmed genomic instability in DU145 cells as PMS2 protein expression was lacking. In fact, they observed this gene to be deficient and correlated with genomic instability in a wider range of PCa cell lines compared to other MMR genes. Martin et al. [18] also found no PMS2 protein in these DU145 cells. A prior study from our laboratory [19] showed the DU145 and LNCaP cell lines to have none to low levels of PMS2 protein expression and these had reduced DNA repair activity as compared to the MMR proficient Hela cells.

PMS2 expression has also been studied in prostate tissue. Reports by Chen et al. [17, 20] found that in the normal prostate gland region, PMS2 protein was predominantly detected in the nuclei of glandular luminal epithelium, basal cells, and some stromal cells. This pattern of PMS2 expression was also observed in the normal adjacent region of prostate tumor tissue. In malignant prostate cells however, PMS2 levels were found to be less than that observed in normal adjacent areas and in fact, more tumor foci showed loss of PMS2 compared to other MMR genes. Other studies also showed reduced PMS2 protein expression in prostate tumor regions as compared to normal adjacent [20, 21]. Additionally, PMS2 expression including both RNA [21] and protein [21, 22] were found to be much lower in PCa when compared to benign prostatic hyperplasia (BPH) tissue; and a significant inverse correlation between Gleason score and PMS2 expression was observed in PCa, making it a good marker of progression [21, 22]. In fact, PMS2 protein expression was observed to be the most downregulated in PCa among the MMR proteins [21].

The PMS2 gene is thus shown to be reduced in PCa cell lines and tissues. Therefore in this study, we characterized the functional role the PMS2 gene plays in PCa cells. Our results are the first to show that re-expressing the PMS2 gene in PCa cells causes inhibition of cell growth both *in vitro* and *in vivo*. Also, we demonstrate PMS2 to enhance apoptosis and induce TMS1 and suppress BCL2A1 genes as a possible mechanism for its protective role in PCa cells.

## RESULTS

### Prostate cancer cell line DU145 is PMS2 deficient

**Figure 1:**
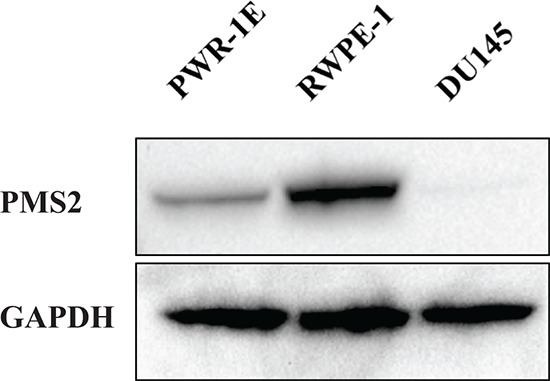
PMS2 expression is downregulated in DU145 PCa cells Prostatic cell lines were grown in culture dishes for two days and underwent Western analyses. Shown is a representative immunoblot of PMS2 protein expression in DU145 and normal prostate epithelial PWR-1E and RWPE-1 cells. GAPDH was used as loading control.

Prior studies have established DU145 cells to be PMS2-deficient [17–19] and thus, we selected this cancerous cell line for further studies. Initially, we confirmed protein expression levels of PMS2 in DU145 cells and as a comparison, measured expression in the normal prostate epithelial cell lines, PWR-1E and RWPE-1. Figure 1 show no protein detected for PMS2 in DU145 cells whereas levels were high in RWPE-1 and low in PWR-1E.

### Effect of PMS2 re-expression on cell proliferation, migration and invasion

**Figure 2:**
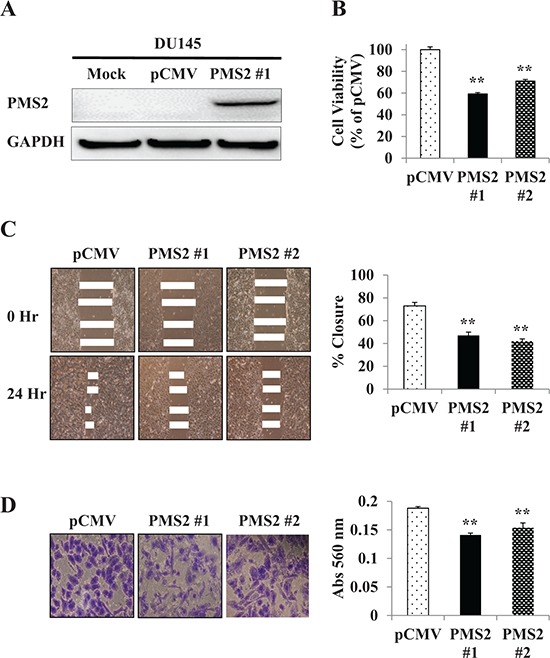
Re-expression and tumor suppressive effect of PMS2 on DU145 cells **A.** Ectopic expression of PMS2. DU145 cells stably transfected with either PMS2 or empty vector (pCMV) along with mock (parental DU145 cells treated with transfection reagent alone) were grown for 48 hours and underwent Western analyses. GAPDH was used as loading control. *Note: Clone #2 (not shown) displayed a band similar in intensity to #1*. **B.** Cell proliferation as analyzed by the MTS cell proliferation assay 72 hours after plating cells. Results are expressed as % and normalized to pCMV control. **C.** Cell migration as measured by wound healing assay. A wound was formed by scraping culture dishes using a pipet tip and closure measured after 24 hours. *Left*: Representative images of wound healing assay are shown. *Right*: Migration expressed as % closure of wound. **D.** Cell invasiveness as measured using Matrigel. Cells were placed onto transwell membrane and allowed to invade for 24 hours. *Left*: Representative images of invading cells are shown. *Right*: Cell invasiveness as measured by absorbance (Abs) at 560 nm. Data are presented as mean ± SEM of at least three experiments; **P < 0.01 PMS2 versus pCMV.

Absence of PMS2 protein in DU145 cells led us to examine whether re-expressing PMS2 affects the growth of these cells. To do this, we established DU145 cell lines that stably express PMS2. As shown in Figure 2A, PMS2 protein was detected by Western blotting in stable PMS2-transfected DU145 cells (clone 1) but not in mock or vector control cells. A second clone also demonstrated a protein band (not shown). To determine the effects of PMS2 on cellular properties, we conducted various functional analyses using both clones. MTS assay showed that cell proliferation was reduced 41% in clone #1 and 29% in clone #2 after 72 hours in DU145 cells expressing PMS2 compared to vector control (*P* < 0.01) (Figure 2B). In accordance, silencing normal PWR-1E cells with two PMS2 siRNAs caused increased proliferation (*P* < 0.08) (Supplementary Figure 1). Wound healing assay demonstrated significant inhibition of cell migration in PMS2-stable compared with vector-transfected DU145 cells after 24 hours (47% closure clone #1 and 42% closure clone #2 versus 73% closure for control, *P* < 0.01) (Figure 2C). Matrigel invasion assay also showed that the number of invading cells was significantly decreased in PMS2 transfectants with absorbance at 560 nm of 0.14 (clone #1) and 0.15 (clone #2) compared with vector control having absorbance of 0.19 (*P* < 0.01) (Figure 2D). These results indicate PMS2 to play an important role in reducing tumor cell proliferation, migration and invasion.

### Effect of PMS2 expression on tumorigenicity *in vivo*

**Figure 3:**
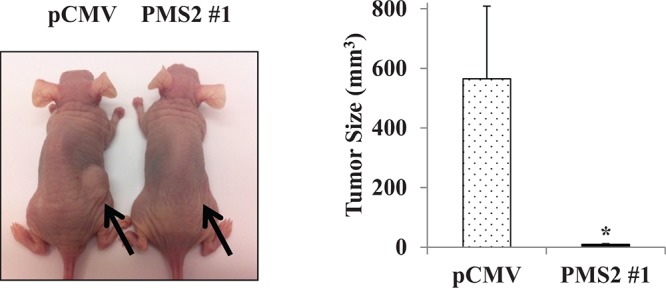
Expression of PMS2 suppresses growth of DU145 cells *in vivo* Athymic nude mice were injected subcutaneously with stable PMS2 or pCMV-transfected DU145 cells and growth determined after 35 days. *Left*: Representative image of tumors in mice. *Right*: Tumor size (mm^3^). Data are presented as mean ± SEM of five mice per group; *P < 0.05 PMS2 versus pCMV.

To validate the suppressive effect of PMS2 under *in vitro* conditions, we also determined effects of PMS2 on tumor growth in animal models. Stable PMS2 and pCMV DU145 cells were subcutaneously injected into nude mice. We observed that expression of PMS2 inhibited DU145 cell tumor formation throughout the duration which lasted 5 weeks whereas tumors grew rapidly and were visible in pCMV animals (*P* < 0.05) (Figure 3). These results suggest PMS2 suppresses PCa cell growth *in vivo*.

### PMS2 influences cellular apoptosis

**Figure 4:**
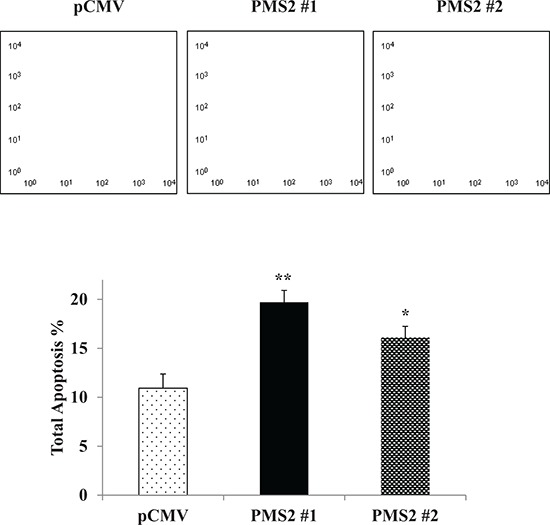
PMS2 expression upregulates apoptosis Stable PMS2-expressing and vector control DU145 cells were grown for 72 hours and apoptosis was measured by flow cytometric analyses. *Top*: Representative biparametric histogram showing cell population in early (bottom right quadrant) and late (top right quadrant) apoptotic, and viable (bottom left quadrant) states for each treatment. *Bottom*: Total apoptosis %. Bar graph is mean ± SEM of three experiments; **P < 0.01, *P < 0.05 PMS2 versus pCMV.

Since PMS2 restoration significantly inhibited cell growth and progression of DU145 cells both *in vitro* and *in vivo*, we hypothesized that its expression may induce apoptosis. Figure 4 shows that the apoptotic and early apoptotic fractions (upper right and lower right quadrants of biparametric histograms, respectively) were increased in PMS2-transfectants compared to vector control after 72 hours of growth. Total apoptosis levels were 10.9% versus 19.7% (*P* < 0.01) and 16.1% (*P* < 0.05) for pCMV versus PMS2-expressing clone #1 and #2 cells, respectively (Figure 4).

### PMS2 induces TMS1 and inhibits BCL2A1 genes

**Figure 5:**
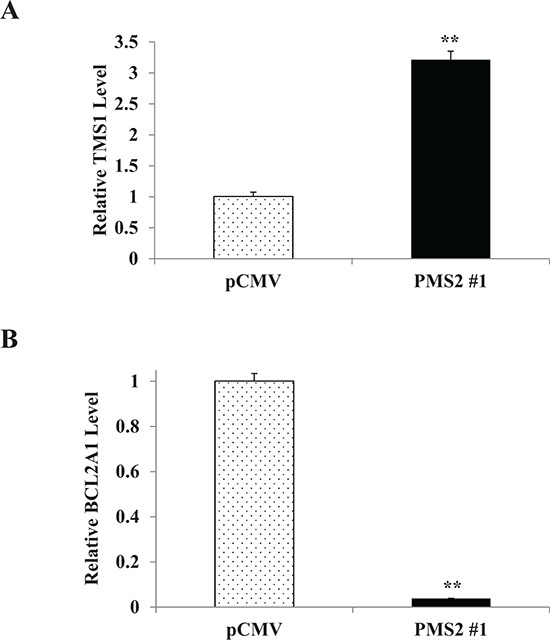
PMS2 induces TMS1 and inhibits BCL2A1 expression in DU145 cells Stable PMS2-expressing and vector control DU145 cells were grown and expression of **A.** TMS1 and **B.** BCL2A1 was determined by real-time PCR. Levels are normalized to vector control. Data are presented as mean ± SEM of three experiments; **P < 0.01 PMS2 versus pCMV.

The pro-apoptotic role for PMS2 observed suggests that apoptotic pathways are affected. We thus performed array analyses to determine which apoptotic genes are altered due to PMS2 in DU145 cells. Table 1 displays genes that are elevated or reduced two-fold or greater. Up-regulation of the Target of Methylation induced Silencing (TMS1) gene due to PMS2 was confirmed by real-time PCR using TaqMan probes as a 3.2-fold increase was observed relative to pCMV controls (*P* < 0.01) (Figure 5A). On the contrary, a reduction of over 90% of B-cell lymphoma 2-related protein A1 (BCL2A1) due to PMS2 was confirmed (*P* < 0.01) (Figure 5B). Other genes did not differ however, between pCMV and PMS2 when analyzed by real-time PCR. These results indicate TMS1 and BCL2A1 to moderate the apoptotic effects of PMS2 in PCa cells.

**Table 1:** Apoptosis-related genes increased or decreased 2-fold or greater due to expression of PMS2 in DU145 clone #1 cells

Gene	Fold change
TMS1	5.3262
IGF1R	2.1167
HRK	2.0917
CD27	2.083
BCL2A1	−3.3694
CASP1	−5.9844
CASP4	−6.6332

## DISCUSSION

The MMR system recognizes and corrects mis-incorporated nucleotides and insertion/deletion mis-pairs formed during DNA replication [9, 10]. Loss of proteins critical in this repair process however, can result in genetic alterations that may ultimately lead to cancer. MMR deficiency also predisposes cells of the body to an increased frequency of inactivating mutations in genes important for suppressing carcinogenesis [2]. Besides their established role in DNA repair, MMR genes are also involved in cell cycle checkpoints and apoptosis stimulated by DNA damage [3, 4]. Resistance to apoptosis and cell death were shown in cells deficient in MMR genes when treated with DNA damaging chemotherapeutic agents [23, 24]. The MMR system is thus vital for maintaining cell integrity and disease prevention.

Among the various MMR genes, the PMS2 gene has been relatively understudied in relation to cancer risk [25]. This gene however, has been shown to play an important role in cellular and repair activity as its deficiency has been shown to cause various aberrations. Early studies in S. cerevisiae find repair of mismatches to be impaired due to mutant PMS gene [26]. Transfection of dominant negative fragment of PMS2 into microsatellite stable cells caused an increase in microsatellite instability [20]. In PMS2-deficient transgenic mice, a much higher rate of mutation was observed after exposure to ionizing radiation [27] as well as thymic lymphomas after treatment with MNU [28] when compared to wildtype animals. A protein truncate leading to deficiency of PMS2 caused a lack of repair function in breast cancer cells [29]. In Hela variants lacking PMS2 protein, extracts were ineffective in correcting base mispair but upon introducing wildtype chromosome 7 into cells, correction of the repair defect was restored [30]. In endometrial cancer line HEC-1-A, PMS2 was observed lacking and defective in correcting a variety of mismatches [31] and transfection of PMS2 caused these cells to increase microsatellite stability and repair of mismatches [32]. In glioblastoma cells resistant to temozolomide treatment, a reduction in PMS2 protein was observed and knockdown of PMS2 in parental cells conferred this resistance [33]. The genetic status of PMS2 in cells thus has high impact in cellular function.

In prostate tissue, PMS2 has been shown to be downregulated in tumor regions as compared to normal areas [17, 20, 21] and benign hyperplasia [21, 22]. Given that PMS2 can affect cell growth parameters, we explored the functional significance the PMS2 gene has on PCa cells which has never been done. PMS2 protein expression in the DU145 cell line has previously been shown to be downregulated [17–19] and our results confirmed this lack of expression. The nature of the loss of PMS2 in this cell line is not known. A comprehensive analysis of MMR mRNA transcripts reported in databases and the literature by Thompson et al. [34] found several alternative forms of PMS2 that could lead to protein of varying lengths. It was demonstrated that in breast, nonsense mutations leading to truncate PMS2 protein occurred in the neoplastic transformation of epithelial cells *in vitro* [29] and tissue specimens [35] and interestingly, truncating mutations have been shown to nullify expression of PMS2 protein [36]. The deficiency of PMS2 in DU145 also impacts cellular genetics as Boyer et al. [16] and Yeh et al. [19] reported reduced MMR activity, and Boyer et al. [16] observed microsatellite instability in these cells. Thus, DU145 PCa cells are an ideal cell line to determine functional effects of PMS2 by gene re-expression.

By expressing the PMS2 gene in DU145 cells, we observed that this gene caused a significant reduction in proliferation, migration, and invasive properties of this PCa cell line. A similar effect was observed *in vivo* as PMS2-expressing cells inhibited tumor growth. This repressive activity was found to be due to apoptosis as PMS2 cells had a much higher rate compared to that of vector control. The apoptotic environment was also supported by an observed rise in TMS1 gene, suggesting the presence of caspase proteins [37] as well as reduction of BCL2A1, indicating the release of pro-apoptotic BCL2-related and BH3-only proteins [38]. Thus, PMS2 plays a tumor suppressor role in PCa cells and this effect is substantiated by enhanced proliferation due to silencing of this gene as observed in normal PWR-1E cells. In concordance with our results, PMS2 has been shown by others to play an important role in apoptosis. Transfection of PMS2 in Hela cells caused a dramatic increase of cisplatin-induced apoptosis and interestingly, caspase-3 activity was significantly increased in these cells [39]. In animal models, treating PMS2 null mice with NMNU or temozolomide caused a reduced apoptotic response compared to wildtype [40] and Zeng et al. [41] observed cells derived from null mice to have reduced levels of apoptosis after infrared exposure, with effect being independent of p53. Furthermore, in adenocarcinoma specimens from patients, those with reduced PMS2 expression had lower apoptotic ability in nearby tissue samples [42].

The pathway by which apoptosis is induced by PMS2 in PCa cells may be through TMS1 as this gene was observed to be the most upregulated. TMS1 consists of two protein interacting domains which are a pyrin domain (PYD) and a caspase recruitment domain (CARD) [43]. This gene is a tumor suppressor and can cause apoptosis by interacting with proteins such as BAX, BID, p53, and caspases [37]. The TMS1 gene is highly methylated with transcriptional repression in various cancers [37]. In prostate, TMS1 is observed to be downregulated in high grade PIN and cancer compared to BPH and normal controls [44], and this reduction has been shown to be due to promoter methylation [45, 46]. In prostatic cell lines, a similar tendency exists as TMS1 expression is silenced in cancer cells including DU145 and methylated, whereas in PrEC (primary prostate epithelial cells), benign BPH-1, and normal RWPE-1 cells, TMS1 is highly expressed and unmethylated [45–47]. Since TMS1 was observed to be upregulated in PMS2-treated DU145 cells, it is thus apparent that PMS2 is capable of reversing the methylation status and inducing expression of TMS1 and warrants further investigation.

In addition to TMS1, apoptosis due to PMS2 may also be caused by downregulation of BCL2A1. BCL2A1 is an anti-apoptotic gene that affects the intrinsic apoptotic pathway. BCL2A1 binds and suppresses various apoptotic agents such as BAK, BID, BIM, PUMA and NOXA [38] and thus, reduction of this gene can result in discharge of these factors culminating in apoptosis. Various cancers such as gastric [48] stomach [48], colon [48, 49], and breast [49] have been shown to have elevated levels of BCL2A1. In prostate, tissue expression levels of BCL2A1 were observed to be increased in cancerous versus normal [49], metastatic versus non-metastatic [50], high versus low stages [50], and was undetectable in BPH [50]. Interestingly in cell lines, BCL2A1 was also highly expressed in cancerous cells that include DU145 [50]. Thus, downregulation of BCL2A1 by PMS2 observed in our study strongly suggests PMS2 to contribute to the apoptotic effect of PCa cells; and this effect may be in concert with the upregulation of TMS1.

In summary, this is the first report that shows PMS2 to display a protection against PCa by inhibiting cell proliferation, migration, and invasion *in vitro* as well as tumor growth *in vivo*. PMS2 induced apoptosis and this was supported by a rise in TMS1 and fall in BCL2A1. Thus, we conclude that PMS2 plays a tumor suppressor role by enhancing apoptosis in PCa cells.

## MATERIALS AND METHODS

### Cell lines and reagents

Human PCa cell line DU145 and non-malignant epithelial prostate cell lines PWR-1E and RWPE-1 were purchased from American Type Culture Collection (Manassas, VA). Keratinocyte serum-free medium, bovine pituitary extract and human recombinant epidermal growth factor were purchased from Invitrogen (Carlsbad, CA). RPMI 1640, Opti-minimum essential medium and penicillin/streptomycin were obtained from the UCSF Cell Culture Facility (San Francisco, CA). Fetal bovine serum (FBS) was a product of Atlanta Biologicals (Lawrenceville, GA).

### Cell culture

DU145 cells were cultured in RPMI 1640 medium supplemented with 10% FBS. PWR-1E and RWPE-1 cells were cultured in keratinocyte growth medium supplemented with 5 ng/mL human recombinant epidermal growth factor and 0.05 mg/mL bovine pituitary extract. All cell lines were maintained at 37°C in a humidified atmosphere composed of 5% CO_2_ and 95% air.

### Western blot analysis

Whole cell extracts from cultured cells were prepared using radioimmunoprecipitation assay buffer (Thermo Scientific, Rockford, IL) containing protease inhibitor cocktail (Roche Diagnostics, Basel, Switzerland). Protein quantification was done using a BCA protein assay kit (Thermo Scientific) according to the manufacturer's instructions. Total protein (20 μg) was loaded onto 4–12% bis–tris gels with 3-(*N*-morpholino) propanesulfonic acid buffer and separated by a NuPAGE electrophoresis system (Invitrogen). Protein was transferred to Invitrogen™ polyvinylidene difluoride and immunoblotting was carried out according to standard protocols. Monoclonal antibody against PMS2 was purchased from Origene Technologies (Rockville, MD) and monoclonal antibody against GAPDH (Santa Cruz Biotechnology, Santa Cruz, CA) was used to confirm equal loading. The membrane was washed and then incubated with secondary antibodies conjugated to horseradish peroxidase (Cell Signaling Technology). Protein complexes were visualized with the Echo-chemiluminescence (ECL) Detection System (GE Healthcare, Little Chalfont, UK) using the Chemidoc Imaging System (Bio-Rad Laboratories, Hercules, CA).

### Real-time quantitative polymerase chain reaction

Total RNA was extracted from DU145, PWR-1E and RWPE-1 cells using the RNeasy Mini kit (Qiagen, Valencia, CA) according to the manufacturer's instructions. Extracted RNA was reverse-transcribed into complementary DNA (cDNA) using iScript cDNA Synthesis kit (Bio-Rad) and TaqMan MicroRNA Reverse Transcription kit (Applied Biosystems, Foster City, CA). Quantitative real-time PCR analysis was performed with an Applied Biosystems Prism 7500 Fast Sequence Detection System using TaqMan Universal PCR master mix according to the manufacturer's protocol (Applied Biosystems). Levels of RNA expression were determined using the 7500 Fast System SDS software version 1.3.1 (Applied Biosystems). PCR parameters for cycling were as follows: 95ºC for 20 seconds, then 40 cycles of 95ºC for 3 seconds and 60ºC for 30 seconds. All reactions were done in a 10 μL reaction volume in triplicate. The data were analyzed using the delta-delta Ct method to calculate the fold-change. TaqMan probes and primers for PMS2 (assay ID: Hs00241053_m1), TMS1 (Hs01547324_gH), BCL2A1 (Hs00187845_m1), and GAPDH (Hs02758991_g1) were obtained from Applied Biosystems. GAPDH was used as internal control. For array analyses, expression of apoptosis-related genes was determined using the Human Apoptosis RT2 Profiler PCR Array (Qiagen) per manufacturer's instructions.

### Establishment of DU145 cells stably expressing PMS2

DU145 cells were transfected with pCMV6-ENTRY vector expressing the C-terminally Myc and Flag-tagged human PMS2 cDNA as well as empty pCMV6-ENTRY vector as a control (Origene) using X-treme Gene HD transfection reagent (Roche Diagnostics, Indianapolis, IN) according to the manufacturer's protocol. Clones were selected using 500 μg/ml of G418 (Invitrogen). Colonies resistant to G418 appeared within 2 weeks and single colonies were picked and then expanded for another 3 weeks to make stable clone stock cells.

### Small interfering RNA transfection

Cells grown to 30–50% confluence were treated with two different sets of small interfering RNA (siRNA) duplexes specific for human PMS2 (siPMS2) or negative control siRNA (Life Technologies) by transfection using Lipofectamine RNAiMAX transfection reagent (Life Technologies) as described by the manufacturer. Knockdown efficiency was evaluated by real-time PCR for the two siPMS2s at different concentrations and the optimal condition selected was 10 nM.

### Cell proliferation assay

An MTS based assay was utilized to determine cell proliferation. Cells were plated in triplicate in 96-well microplates at a density of 5 × 10^3^ cells per well. At the desired time point, the number of viable cells was determined by adding CellTiter 96 AQueous One Solution reagent (Promega, Madison, WI) to each well and measuring the absorbance at 490 nm on a SpectraMax 190 plate reader (Molecular Devices, Sunnyvale, CA). Results were expressed as the percentage of optical density with absorbance of control cells being 100%.

### Migration and invasion assays

Cell migration was evaluated by a wound-healing assay. Cells were plated in six-well dishes and monolayers were scraped using a P-20 micropipette tip. The width of the initial gap (0 hour) and the residual gap 24 hours after wounding were calculated from photomicrographs taken using a Nikon Eclipse TS100 microscope (Technical Instruments, Burlingame CA). Cell invasion properties were measured by a procedure using the CytoSelect Invasion assay (Cell Biolabs, San Diego CA) along with modified Boyden chambers consisting of transwell-precoated Matrigel membrane filter inserts with eight micron pores in 24-well tissue culture plates (BD Biosciences, Bedford, MA). Minimum essential medium containing 10% FBS in the lower chamber served as the chemo-attractant. After 24 hours, invasive cells were stained and observed by photomicrographs taken with the Nikon microscope and quantified using the SpectraMax plate reader at absorbance of 560 nm.

### *In vivo* tumor growth

All animal care was in accordance with the guidelines and this study was approved by the San Francisco Veterans Affairs IACUC (Institutional Animal Care and Use Committee). Animal users completed training programs to handle and work with mice through AALAS (American Association for Laboratory Animal Science) prior to animal experiments. For the subcutaneous xenograft mouse model, DU145 cells (5 × 10^6^) stably transfected with PMS2 or empty pCMV6-ENTRY vector were suspended in 100 μL RPMI 1640 medium and subcutaneously injected into the right backside flank of five week old female nude mice (strain BALB/c nu/nu; Charles River Laboratories, Wilmington, MA). Five nude mice were used per treatment group and tumor growth was examined after 35 days. Tumor volume was calculated on the basis of width (x) and length (y) using the formula: x^2^y/2, where x < y.

### Apoptosis assay

Apoptosis was analyzed with an annexin V-fluorescein isothiocyanate (FITC)/7-amino-actinomycin D staining system obtained from BD Biosciences (San Diego, CA). Briefly, prostate cells were harvested and resuspended in binding buffer at a concentration of 1 × 10^6^ cells/ml. For each assay, 1 × 10^5^ cells were incubated with 5 μl of annexin V-FITC and 5 μl of 7-amino-actinomycin D in the dark for 15 min at room temperature. After adding 400 μl of binding buffer, samples were analyzed within an hour by a Cell Lab Quanta™ SC MPL flow cytometer (Beckman Coulter, Fullerton, CA).

### Statistical analyses

Values in Figures are presented as the mean ± standard error of mean (SEM) based on results obtained from at least three independent experiments. For *in vivo* studies, results are based on five animals per group. The relationship between variables was analyzed using the non-parametric Mann-Whitney *U* test or two-tailed Student's *t*-test. All analyses were performed using Expert StatView (version 4, SAS Institute, Cary, NC).

## SUPPLEMENTARY FIGURE


