# Supplementary material for: Functional role of DNA mismatch repair gene PMS2 in prostate cancer cells
Source: Oncotarget. 2015 May 6;6(18):16341–51. doi: 10.18632/oncotarget.3854 (PMC4599273; doi:10.18632/oncotarget.3854)
Supplement: Supplementary file 1 [file oncotarget-06-16341-s001.pdf]

SUPPLEMENTARY FIGURE

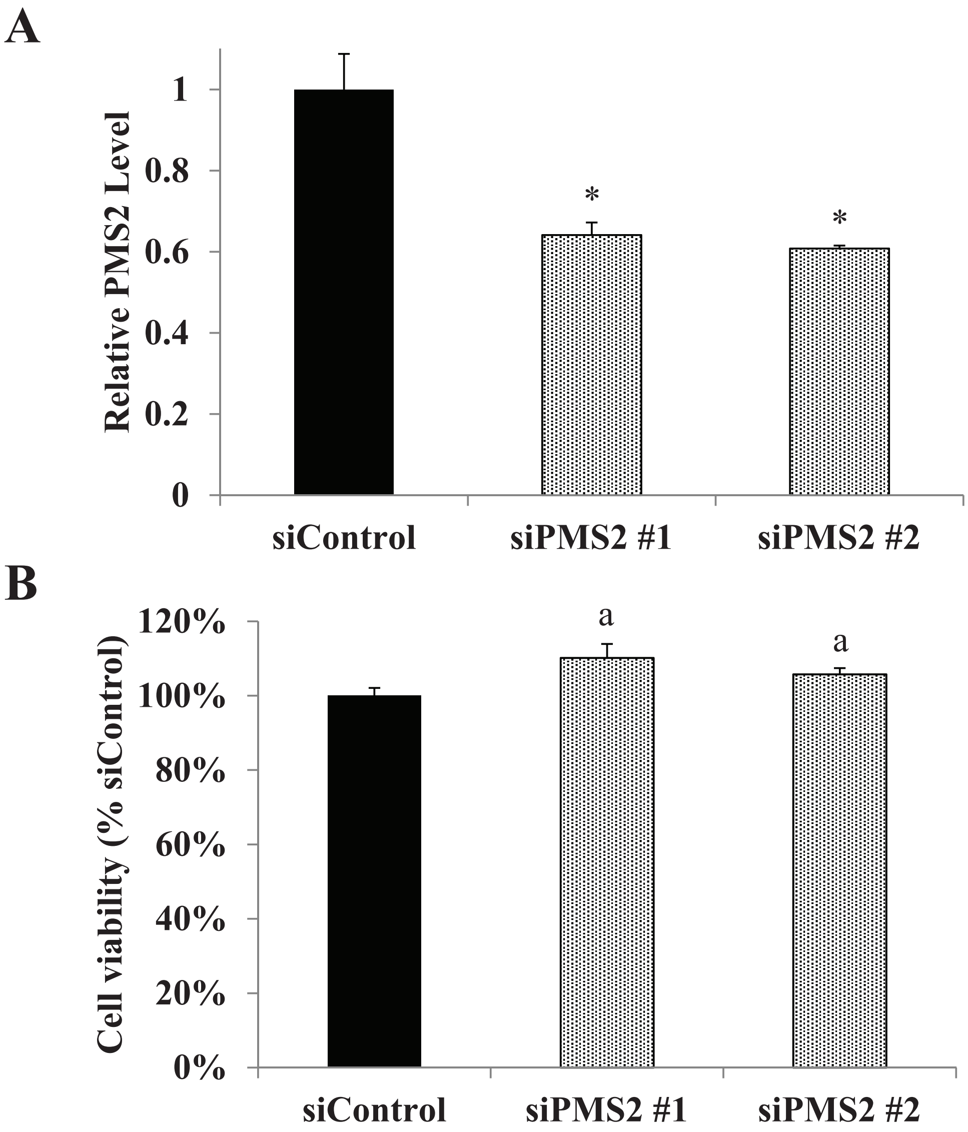

Supplementary Figure S1: PMS2 knockdown causes increased proliferation of normal prostatic cells. PMS2 siRNAs (siPMS2s) were transfected individually along with non-specific siRNA control (siControl) into PWR-1E cells for 48 hours. **A.** Expression was analyzed by real-time PCR using TaqMan probe for PMS2. **B.** Proliferation was analyzed by the MTS cell proliferation assay. Data are presented as mean ± SEM of three experiments and normalized to siControl; $^{*}P < 0.05$, $^{a}P < 0.08$ siPMS2 versus siControl.
